# Quality Assessment in Dobutamine Stress Echocardiography: What are the Clinical Predictors Associated With a Non-Diagnostic Test?

**DOI:** 10.4021/cr154w

**Published:** 2012-03-20

**Authors:** Katie M. Hawthorne, Amer M. Johri, Rajeev Malhotra, Judy Hung, Aaron Baggish, Michael H. Picard

**Affiliations:** a Division of Cardiology and Department of Medicine, Massachusetts General Hospital and Harvard Medical School, Boston MA, USA; b Authors contributed equally to writing of manuscript

**Keywords:** Stress echocardiography, Non-Diagnostic, Coronary artery disease

## Abstract

**Background:**

Non-diagnostic dobutamine stress echocardiography (ndDSE, failure to achieve 85% of maximal predicted heart rate (HR) without evidence of inducible ischemia) is an important limitation affecting quality of DSE testing. The objectives of this study were to identify the clinical variables associated with a non-diagnostic Dobutamine Stress Echocardiogram (ndDSE) and further evaluate the patterns of subsequent testing for myocardial ischemia.

**Methods:**

Consecutive DSE’s over a 17 month period (January 2008 to June 2009) were studied. Baseline demographics, medical history, and vital signs were collected. Subsequent testing was determined for up to 6 months after the initial DSE. Univariate and multivariate logistic regression analysis was performed to identify clinical factors associated with ndDSE.

**Results:**

Of 467 total DSE, 314 (67%) were negative for ischemia, 69 (15%) were positive, and 84 (18%) were ndDSE. Of those recommended for further nuclear MPI testing 12 (14%) had an ndDSE compared to 16 (4%) patients with a diagnostic DSE (P = 0.001). Fifty percent of the ndDSE nuclear MPI tests were positive for ischemia. In the univariate analysis, Diabetes Mellitus (DM; P = 0.003), calcium channel antagonist (CCA) use (P = 0.047), Hypertension (HTN; P = 0.06), low baseline HR (P < 0.001), and younger age group (P = 0.02) were predictive of ndDSE. Of these, all except CCA use remained independent predictors of ndDSE in multivariate analysis. A 4 variable model for predicting ndDSE was developed from the multivariate logistic regression displayed in Table 1 (age and baseline HR were categorized and scored 0-2; DM and HTN were scored as 0 (absent) or 1 (present)). Figure 2 demonstrates how risk of ndDSE correlated with a higher score, with each increment having an odds ratio of 2.1 (P < 0.001).

**Conclusions:**

DM, HTN, younger age, and lower baseline HR affect the quality of DSE testing, resulting in non-diagnostic tests. A model combining these factors can identify patients most likely to have this outcome. Identification of this cohort may improve referral patterns and improve the quality of stress testing.

## Introduction

In this era of cost-control and burgeoning cardiac risk factors, health professionals and organizations are placing greater emphasis on continuous quality improvement of cardiac testing, especially of cardiac imaging. Within the field of echocardiography, initiatives have been implemented to improve quality by developing methods to reduce inter- and intra-observer variability, develop appropriateness criteria, and design protocols that reduce non-diagnostic outcomes that otherwise necessitate repeat or further testing [1, 2]. For example, Appropriate Use Criteria (AUC) are constantly being updated in the field of Stress Echocardiography from the original 2008 AUC report released by the American College of Cardiology Foundation, ensuring proper administration of stress testing based on the results of clinical data and through reflection of patient outcomes [3].

Dobutamine Stress Echocardiography (DSE) is a commonly used non-invasive imaging technique for the diagnosis of cardiovascular disease and subsequent risk assessment [4, 5]. However, the major challenge in obtaining a diagnostic result is the ability to achieve a target heart rate (HR) of at least 85% of maximal predicted heart rate (MPHR) [6]. By convention, a DSE is considered non-diagnostic if the patient fails to achieve 85% of MPHR in the absence of an inducible wall motion abnormality (WMA) [7]. Non-diagnostic DSE (ndDSE) is common and may lead to additional testing for myocardial ischemia [8], thereby further contributing to the increasing costs of cardiac care. Suboptimal images also routinely lead to non-diagnostic stress test outcomes, with previous studies reporting this problem in as many as 1 of 3 (33%) stress echocardiograms by routine two-dimensional methods [9, 10]. While techniques such as contrast agent use in stress echocardiography were shown to help reduce the number of non-diagnostic outcomes through improvement of image quality, understanding the baseline clinical characteristics associated with a non-diagnostic test may aid in selecting appropriate patients for DSE and in providing alternative testing mechanisms to those identified as being at high risk of a non-diagnostic result. The objectives of this study were to: 1) determine the prevalence of ndDSE in patients undergoing evaluation of myocardial ischemia; 2) evaluate the pattern of subsequent testing for myocardial ischemia following a diagnostic DSE versus ndDSE; and 3) identify the clinical variables associated with ndDSE.

## Methods

### Study design

The Massachusetts General Hospital (MGH) echocardiography database (January 2008 to June 2009) was reviewed to identify DSE performed for diagnosis of Coronary Artery Disease (CAD). Patients were excluded if complete data were not available, if identification of CAD was not the assessment criterion (e.g., hypertrophic cardiomyopathy evaluation, valvular lesion assessment, etc.) or if testing was performed while on beta-blocker therapy. Institutional Review Board approval was obtained prior to collection of data from the electronic medical record and subsequent analysis.

### DSE protocol

Following written, informed consent, a standard dobutamine stress test protocol was performed following guidelines of the American Society of Echocardiography [7]. Based on previous work in our laboratory [11, 12], beta blocker therapy was held 24 - 48 hours prior to the DSE, provided the referring physician was in agreement.

Resting and peak stress vital signs, body mass index, resting and peak stress electrocardiographic parameters, patient symptoms, reason for termination (if applicable), and stress test outcome data were recorded.

A negative DSE was defined as a test that achieved at least 85% of MPHR and had no new WMA noted. A positive DSE was defined as a DSE that was found to have new WMA, regardless of HR achieved. A ndDSE was defined as failure to achieve 85% MPHR, without evidence of inducible WMA at this submaximal HR.

### Data collection

Data was collected by a single investigator (KH) from the electronic medical records and included: patient demographics, medical history, medication use, and indication for stress echocardiography referral. The electronic medical record was also used to determine the pattern of further non-invasive testing following the index DSE. Left heart catheterization rate following the initial DSE was additionally determined. Only the first test conducted (either nuclear myocardial perfusion imaging (MPI) or cardiac catheterization) as a follow up to the DSE was included for the purposes of our study. A follow-up study was considered positive if a nuclear MPI was interpreted as demonstrating myocardial ischemia or coronary angiography revealed stenosis of greater than 50%.

### Statistical analysis

Statistical tests were performed using the STATA 8.0 software package (StataCorp LP, College Station, TX). Normality of data was assessed using the Shapiro-Wilk test. Continuous variables were expressed with either mean ± SD or median (interquartile range, IQR), as appropriate (or as determined by the Shapiro-Wilk test). Group baseline characteristics were compared using either the Student t test or Mann-Whitney U statistic for continuous variables, as appropriate, or Pearson’s chi square test for categorical variables. Univariate and multivariate logistic regression was performed to identify predictors of ndDSE. A proposed scoring system was developed with components weighted based on the odds ratios obtained from the multivariate regression analysis. A P-value of < 0.05 was considered significant.

## Results

### Prevalence of a non-diagnostic test

**Figure 1:**
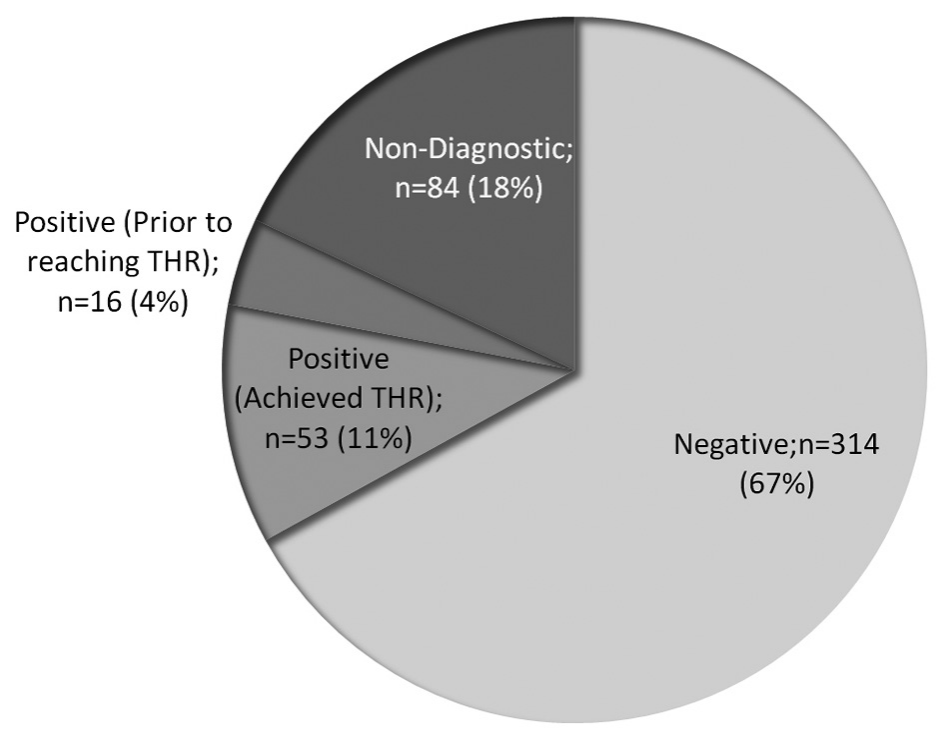
Frequency of dobutamine stress echocardiography test outcomes in patients evaluated for ischemia (n = 467). THR, target heart rate.

Of the 533 patients that were referred for DSE during the study period, 4 patients were excluded from analysis due to incomplete data from the electronic medical record, 57 patients were excluded for a non-CAD indication, and 5 patients were excluded because beta blocker restriction was not satisfied. Thus 467 patients were included for analysis. Of these, 314 (67%) reached target heart rate and were negative for ischemia, 69 (15%) were positive, and 84 (18%) were non-diagnostic (Fig. 1).

### Determinants of a non-diagnostic DSE

The measures of association between baseline data and a non-diagnostic test are shown in Table 1. In the univariate analysis, Diabetes Mellitus (DM) (P = 0.003), calcium channel antagonist (CCA) use (P = 0.047), low baseline HR (P < 0.001), and younger age group (P = 0.02) were predictive of ndDSE. The presence of hypertension demonstrated a trend towards predicting ndDSE (P = 0.057). Of these, all except CCA use were independent predictors in the multivariate analysis.

**Table 1:** Baseline Characteristics of Patients With Diagnostic and Non-Diagnostic Dobutamine Stress Echocardiography

Clinical Variable	Diagnostic – Positive or Negative Test (n = 383)	Non-Diagnostic (n = 84)	P-value
Age (mean)	59 ± 12	57 ± 10	0.09
Age Group			0.04
< 60 years	193 (50%)	52 (62%)	
60 - 69 years	118 (31%)	25 (30%)	
≥70 years	72 (19%)	7 (8%)	
Mean Body Mass Index (range)	29 (25 - 33)	28 (25 - 35)	0.67
Mean Baseline Heart Rate, bpm (range)	76 (67 - 85)	70 (65 - 77)	< 0.001
Mean Baseline SBP, mmHg (range)	136 (120 - 157)	144 (121 - 167)	0.07
Mean Baseline DBP, mmHg (range)	71 (63 - 81)	74 (62 - 84)	0.60
Hypertension	276 (72%)	69 (82%)	0.057
Previous Myocardial Infarction	33 (9%)	5 (6%)	0.42
Prior Coronary Artery Disease Procedure (Percutaneous Intervention, Coronary Artery Bypass Graft)	49 (13%)	8 (10%)	0.41
Congestive Heart Failure	45 (12%)	11 (13%)	0.73
Diabetes Mellitus	142 (37%)	46 (55%)	0.003
Peripheral Vascular Disease	52 (14%)	12 (14%)	0.86
Hypercholesterolemia	169 (44%)	39 (46%)	0.70
History of Tobacco Use	206 (54%)	50 (60%)	0.35
Beta-Blocker Use	220 (57%)	47 (56%)	0.80
Angiotensin Converting Enzyme Inhibitor	80 (21%)	24 (29%)	0.13
Angiotension Receptor Blocker	58 (15%)	12 (14%)	0.84
Nitrate	19 (5%)	5 (6%)	0.71
Any Calcium Channel Antagonist Use	100 (26%)	31 (37%)	0.046
Dihydropyridine Calcium Channel Antagonist	76 (20%)	22 (26%)	0.196
Non-dihydropyridine			
Calcium Channel Antagonist	24 (6%)	9 (11%)	0.150
Statin Use	141 (37%)	32 (38%)	0.83
Amiodarone Use	10 (3%)	3 (4%)	0.63

**Figure 2:**
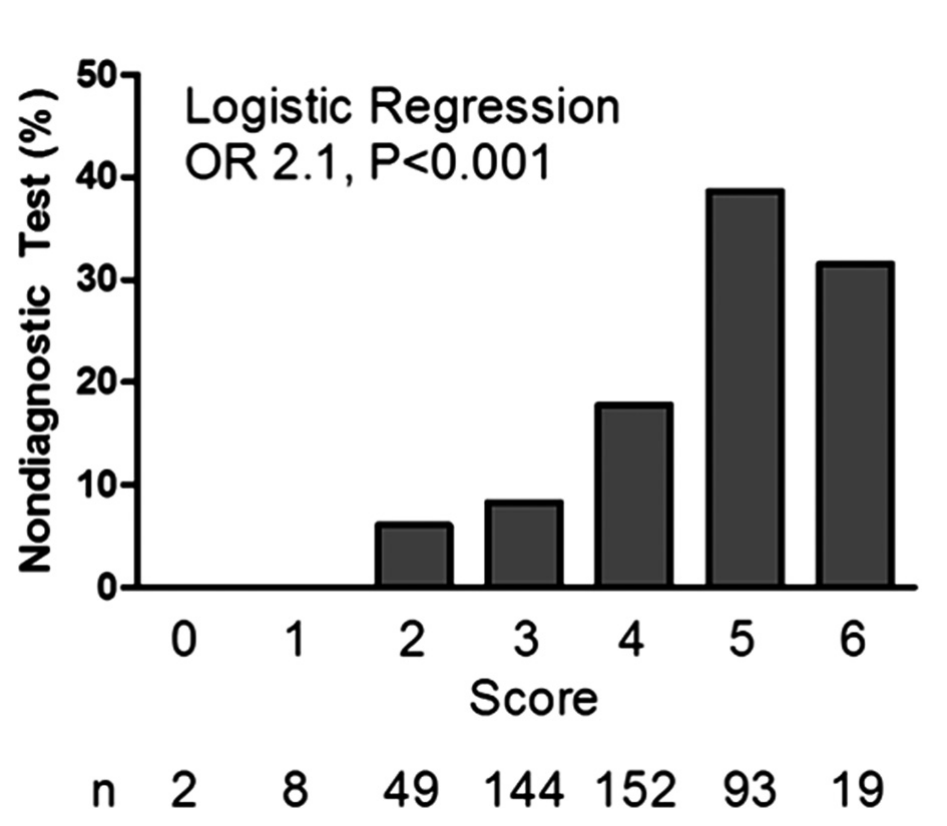
Risk stratification by scoring system: percentage of patients with a non-diagnostic test in each score category (n = 467).

A proposed scoring system from a four variable model for predicting ndDSE was developed from the multivariate analysis (Table 2), incorporating age group (< 60, 60 - 69, and ≥ 70 years), baseline HR group (< 70, 70 - 89, and ≥ 90 bpm), the presence or absence of DM, and the presence or absence of hypertension. Each variable was approximately weighted according to its associated odds ratio. Patients were assigned a point score based upon their age (0: ≥ 70 years; 1: 60 - 69 years; 2; < 60 years) and baseline heart rate (0: ≥ 90 bpm; 1; 70 - 89 bpm; 2: < 70 bpm). Additionally, presence (1) or absence (0) of both DM and HTN were accounted for, providing a total possible score of 0-6 for each patient. Risk of ndDSE correlated with a higher score, with each increment in the score having an odds ratio for ndDSE of 2.1 (P < 0.001) (Fig. 2).

**Table 2:** Predictors of Non-Diagnostic Testing Identified by Multivariate Logistic Regression Analysis With an Associated Scoring System

Clinical Predictor	Multivariate Odds Ratio (95% CI)	P-value	Scoring System
Age Group	0.47 (0.32 - 0.70)	< 0.001	
< 60 years (n = 245)			2
60 - 69 years (n = 143)			1
≥ 70 years (n = 79)			0
Baseline Heart Rate	0.41 (0.28 - 0.62)	< 0.001	
< 70 bpm (n = 167)			2
70 - 89 bpm (n = 225)			1
≥ 90 bpm (n = 75)			0
DM	1.91 (1.16 - 3.16)	0.01	
Absent (n = 279)			0
Present (n = 188)			1
Hypertension	1.95 (1.02 - 3.71	0.04	
Absent (n = 122)			0
Present (n = 345)			1
Scoring System	2.12 (1.65 - 2.73)	< 0.001	0-6

### Repeat testing

Of the 467 patients in the study, 93 patients (20%) had further testing within the ensuing 6 months (Table 3). Four percent of patients with a diagnostic DSE (n = 16) and 14% of patients with an ndDSE (n = 12) underwent nuclear MPI testing. The proportion of patients proceeding to cardiac catheterization (14%) was identical in both the non-diagnostic DSE and diagnostic DSE group (Table 3). Of those ndDSE patients that proceeded to subsequent testing, 50% had a positive nuclear MPI test and 58% had a positive cardiac catheterization result for myocardial ischemia.

**Table 3:** Repeat Testing Patterns in Patients With a Non-Diagnostic Vs. Diagnostic DSE (Pearson’s Chi Square Used to Obtain P-Values)

	Non-Diagnostic (n = 84)	Diagnostic (n = 383) Negative (n = 314) Positive (n = 69)	P-value
Nuclear Study	12 (14%)	16 (4%)	P < 0.01
Cardiac Catheterization	12 (14%)	53 (14%)	P = 0.913

## Discussion

Previous studies have quantified side-effects (tachyarrythmias, hypotension, bradycardia, hypertension, headache, nausea) leading to early study termination and thus submaximal or non-diagnostic dobutamine stress tests [13]. Currently, there is interest in developing appropriateness criteria and improving quality of echocardiographic testing. However, before these goals can be achieved, the underlying patient variables that lead to non-diagnostic outcomes must be understood. We observed the prevalence of a non-diagnostic DSE test at our institution was common and similar to findings at other centers, suggesting a systemic set of variables that may be affecting quality of testing. At our institution, we found that DM, CCA use, younger age, and low baseline HR were associated with a non-diagnostic test. A significant portion of these patients required alternative testing, and half of these patients were positive for myocardial ischemia, underlining the fact that this is not a trivial issue. Understanding the baseline variables that may be associated with a non-diagnostic test is required before quality control measures, such as developing appropriateness criteria for stress testing can be implemented.

Although there is a paucity of data on the association between diabetes and –non-diagnostic stress testing, our finding was in agreement with the few published reports [14, 15]. Patel et al. reported a higher rate of diabetes in patients with a negative submaximal DSE compared to those with a negative maximal DSE. Of note, they demonstrated that diabetic patients with a negative submaximal DSE (< 85%MPHR) were more likely to have a cardiac event, emphasizing the need for further evaluation of CAD within this population [14]. Similarly, Ballal et al [15] reported that in diabetic patients with a negative and submaximal DSE, the adverse cardiac event rate was analogous to patients displaying positive results for myocardial ischemia via DSE testing. The cardiac event rate during 28 months of follow up observations within the negative submaximal DSE group was 31%, compared to a 36% event rate in the positive DSE groups.

The association between diabetes and ndDSE may be due to cardiac autonomic neuropathy, a condition that occurs in many diabetic patients. Cardiac autonomic neuropathy within these individuals is the result of complex interactions among which degree of glycemic control, disease duration, age-related neuronal attrition, and systolic and diastolic blood pressure [16, 17] all contribute to cardiac dysfunction. Autonomic innervation is the primary extrinsic control mechanism regulating heart rate variability and cardiac performance. It has been shown that chronic hyperglycemia promotes progressive autonomic neural dysfunction in a manner that parallels the development of peripheral neuropathy.

CCA use as a univariate predictor of ndDSE was also identified. Patients were instructed to hold beta-blocker therapy for forty-eight hours prior to the DSE as these competitive antagonists markedly attenuate the ability of DSE to detect a significant coronary lesion [11, 12]. Further evaluation of withholding CCA use prior to a DSE is warranted based on this univariate prediction result.

Younger age and lower baseline HR were associated with ndDSE in those patients included in the analysis. Both factors require a greater change from baseline HR to achieve 85% MPHR, possibly explaining why these patients failed to achieve the HR required for a diagnostic test result.

Although our study has identified some of the underlying patient characteristics resulting in non-diagnostic testing at our center, implementing a solution to improve overall quality remains a challenge and dependent upon uptake of this information by the referring physician. We are currently investigating the implementation of the proposed scoring system prior to patient arrival in the stress laboratory. Based on the scoring system, patients in the highest strata (score of 5 or 6) have a 38% probability for a non-diagnostic study. Initiating a discussion with the referring physician during screening with the proposed scoring system may reduce the number of non-diagnostic tests and the consideration of a more appropriate alternative test in some situations; the overall goal being the reduction of repeat testing, wait times, and cost, while enhancing the quality of patient care.

## Abbreviations

CAD: coronary artery disease
DSE: dobutamine stress echocardiography
MPHR: maximal predicted heart rate
ndDSE: non-diagnostic dobutamine stress echocardiography
WMA: wall motion abnormality
MPI: myocardial perfusion imaging
DM: diabetes mellitus
CCA: calcium channel antagonist
HR: heart rate
MIBI: perfusion sestamibi stress test
